# Do Self-Regulated Learning Practices and Intervention Mitigate the Impact of Academic Challenges and COVID-19 Distress on Academic Performance During Online Learning?

**DOI:** 10.3389/fpsyg.2022.813529

**Published:** 2022-03-16

**Authors:** Allyson F. Hadwin, Paweena Sukhawathanakul, Ramin Rostampour, Leslie Michelle Bahena-Olivares

**Affiliations:** Department of Educational Psychology and Leadership Studies, University of Victoria, Victoria, BC, Canada

**Keywords:** self-regulated learning (SRL), online learning, COVID-19, post-secondary academic performance, academic challenges, student success

## Abstract

The COVID-19 pandemic introduced significant disruptions and challenges to the learning environment for many post-secondary students with many shifting entirely to remote online learning. Barriers to academic success already experienced in traditional face-to-face classes may be compounded in the online environment and exacerbated by stressors related to the pandemic. In 2020–2021, post-secondary institutions were faced with the reality of rolling out fully online instruction with limited access to resources for assisting students in this transition. Instructional interventions that target students’ ability to self-regulate their learning have been shown to improve academic performance and self-regulated learning (SRL) competencies have also been found to mediate the effect of SRL interventions on higher education. However, few studies have examined the efficacy of fully online SRL intervention on mitigating the impact of psychological distress and academic challenges on academic success. This study examined the moderating roles of self-regulatory practices and SRL intervention in buffering the influence of COVID-related psychological distress and academic challenges on academic outcomes (self-reported grade point average (GPA) and academic challenges) in a Canadian sample of undergraduate students (*n* = 496). We found (a) levels of metacognitive and motivational challenges fully mediated the impact of COVID distress on GPA, (b) SRL adapting practices moderated the impact of metacognitive challenges on GPA, and (c) semester-long SRL intervention buffered the impact of COVID distress on academic challenges and resulted in lower levels of social-emotional, cognitive, and metacognitive challenges for first year undergraduate students.

## Introduction

During the start of the COVID-19 pandemic in 2020, many students faced the additional challenge of transitioning to undergraduate studies delivered fully online by instructors and institutions with limited pedagogical or technological experience delivering fully online instruction. Learning to adapt and respond productively to challenges as they arise during studying is the hallmark of self-regulated learning (SRL). Self-regulating learners are goal directed, optimizing strategy selection and deployment to progress toward goals and adaptively respond to new situations and challenges (Zimmerman, 1989; Winne and Hadwin, 1998; Pintrich and Zusho, 2007). Theory predicts these students should be well poised to respond to challenges in productive ways that reduce the impact of academic challenges on academic success outcomes such as grade point average (GPA). Extant research examining the academic outcomes and experiences of students who receive SRL support indicates SRL skills and competencies can be taught and developed (Jansen et al., 2019; Theobald, 2021). However, a limited body of research to date points to the importance of SRL during online learning (e.g., Broadbent and Poon, 2015). The aim of this study is to examine (a) the role SRL competencies play in mitigating the impact of COVID-19-related psychological distress on academic outcomes, and (b) how SRL competencies and academic challenges differ between students who do and do not receive explicit SRL intervention during a global pandemic where all learning occurred online.

### Factors Contributing to Academic Success

Many factors contribute to academic success. From a self-regulatory perspective, Weinstein and colleagues conceptualized academic success as comprising skill, will and self-regulation (Weinstein, 1994; Weinstein and McCombs, 1998; Weinstein et al., 2000). *Skill* refers to having the metacognitive and conditional knowledge about strategies that is necessary to choose and deploy strategies well-suited to the task, context, and learner characteristics. *Will* focuses on a broad range of motivational and affective constructs involved in directing and sustaining effort and persistence. *Self-regulation* refers to strategic self-management of cognition and learning, behaviors such as time management and help-seeking, motivational and emotional beliefs and experiences, and metacognitive monitoring and control of strategies themselves (Weinstein et al., 2011). Numerous meta-analyses and systematic reviews have collated findings across studies to identify specific psychosocial predictors of academic success and retention (e.g., Robbins et al., 2004; Richardson et al., 2012; Schneider and Preckel, 2017; Saunders-Scott et al., 2018). Extending the skill, will and self-regulation framework, we posit that psychological predictors of academic success can be loosely organized into five categories: cognitive, motivational, metacognitive, behavioral, and social/emotional factors.

*Motivational factors* comprise aspects of will, desire and confidence to exert effort and persist in academic tasks, particularly when they are difficult or challenging. Motivation factors associated with academic success and performance include effort regulation and self-efficacy beliefs. Meta-analyses and systematic reviews consistently identify motivational factors, particularly self-efficacy, as strong predictors of academic outcomes such as GPA and retention (e.g., Robbins et al., 2004; Richardson et al., 2012; Fong et al., 2017; van der Zanden et al., 2018).

*Metacognitive factors* have been described as the awareness and control of mental thoughts (Flavell, 1979). Self-monitoring, planning, and building self-awareness about beliefs and practices related to learning and success have most often been examined as predictors of academic outcomes. Two systematic reviews indicate that metacognition, often conflated with SRL, correlate with GPA in post-secondary settings (Richardson et al., 2012) including online environments (Broadbent and Poon, 2015).

*Cognitive factors* include learning, remembering, communicating and expressing ideas, and comprehending course concepts regardless of the mode of presentation. Typically referred to as academic study skills, these factors include directing and sustaining attention, selecting, and encoding new information, and being able to access or recall that information from memory. Meta-analyses reveal that cognitive factors and academic study skills, contribute to academic outcomes such as GPA (Richardson et al., 2012), and retention (Robbins et al., 2004).

*Behavioral factors* involve structuring the learning environment, tasks, and studying to optimize engagement. Behavioral factors such as time management, environment management, attendance, task structuring and distribution, and procrastination have been found to have both direct and indirect effects on academic performance (Broadbent and Poon, 2015; Schneider and Preckel, 2017; van der Zanden et al., 2018).

*Social and emotional factors* refer to one’s overall psychological, social, and physical well-being including managing emotions such as test anxiety, social belongingness and connectedness with campus life and community, and physical health and wellness such as nutrition, sleep, and physical activity. Aspects of social and emotional well-being have been found to be associated with academic performance (van der Zanden et al., 2018), persistence (You, 2018), and retention (Saunders-Scott et al., 2018).

### Self-Regulated Learning Practices Have Been Associated With Academic Success

Self-regulated learning is a goal-directed process through which students take active and strategic control of their learning. Models of SRL share four fundamental features (c.f, Zimmerman and Schunk, 2011; Schunk and Greene, 2018): (1) Striving toward self-set goals and standards is central to SRL; (2) Metacognitive monitoring and awareness direct learners to when and how to exercise strategic control over learning; (3) Multiple facets of learning are implicated (e.g., motivation, emotions, cognition, and behaviors); (4) Presence of recursive cycles of forethought and planning, strategic engagement, reflection and adaptation.

Numerous self-regulatory practices have been found to contribute to academic success in conventional (Jansen et al., 2019) and online (Broadbent and Poon, 2015) post-secondary contexts. *Task perceptions* (forethought and planning) have been associated with (a) academic success (Callan and Cleary, 2019), task success, and overall GPA (Oshige et al., 2007; Hadwin et al., 2008; Miller, 2009), (b) goal and planning quality (Greene et al., 2012; Beckman et al., 2021), (c) self-efficacy (Miller, 2009), and (d) procrastination and disorganization (Cosnefroy et al., 2018). *Goal setting and planning practices* have been found to contribute to post-secondary academic achievement (Jansen et al., 2019). *Regulation of motivational beliefs and behaviors* has been associated with academic achievement (Kim et al., 2020). Practices for *allocating and controlling time* have been empirically linked to academic outcomes such as GPA (Thibodeaux et al., 2017; Adams and Blair, 2019; Wolters and Brady, 2020).

Theoretically, regulation engages learners in a series of contingent events driving strategic adaptation (Winne and Hadwin, 1998). When learners detect misalignment between goals and progress, they are faced with one of a limited set of options including (a) persist with whatever they were doing and hope it will work better in the future, (b) try a new strategy or approach, (c) adjust or fine tune the strategy, (d) update or change planning in the form of task understanding or goals, or (e) reduce effort or withdraw from the task altogether. Acting in response to detected problems invites a new round of monitoring and evaluating which may in turn lead to continued refinement in approaches. This cyclical process is the essence of strategic and adaptive regulation. Despite the theoretical importance of adaptation and metacognitive control in SRL, student success research has virtually ignored this construct. While metacognitive practices including monitoring, self-evaluation and metacognitive awareness have been found to contribute to academic achievement (e.g., Callan and Cleary, 2019; Colthorpe et al., 2019), practices associated with adapting learning and strategy choices as the result of monitoring and evaluation have been under-examined (Craig et al., 2020). Theoretically adaptation practices deployed when strategies and approaches do not work are essential to SRL (Butler and Winne, 1995; Hadwin and Winne, 2012).

### Navigating the Challenges of Learning Online During a Pandemic

Adjusting to university is not easy for many students. Students are expected to learn and work quite independently with minimal formal instruction about when, how, or what to study (Hadwin and Winne, 2012). Even the most successful high school students report facing new kinds of academic challenges at university. Despite extensive research about the factors contributing to student success, there is extant research examining the specific challenges students report during studying. Koivuniemi et al. (2017) used an open-ended questionnaire to collect data about the types of learning challenges and regulatory skills students deploy across different learning situations. The challenges most frequently reported by students included time management, cognitive strategies, concentration, and tiredness. Student with high SRL skills (as measured by the MSLQ) reported a higher frequency of cognitive challenges during individual learning that students ranking low on SRL skills (Pintrich et al., 1993).

Hadwin et al. (2019a,b) examined challenges reported in a weekly study diary over nine consecutive weeks. Students selected from 12 specific challenges including: motivation/procrastination confidence, goal, and time management, choosing or using strategies, learning and remembering, optimizing conditions for studying, language and communication, adjusting to a new culture, emotions, mental health and well-being and life and self-management, or something else. The most frequently reported challenges were motivation and emotion, goal setting and planning, and cognitive challenges. Motivation and emotional challenges were consistently high, regardless of level of goal attainment. Consistent with findings reported by Koivuniemi et al. (2017), cognitive challenges tended to be more frequently reported among students who were academically successful in terms of attaining goals they set for studying that week.

The abrupt move to fully online instruction introduced new challenges. Many students had limited experience with online learning and were living and learning under new stressors associated with pandemic. In addition to academic challenges, the pandemic also introduces significant social and emotional challenges that can indirectly impact students’ academic performance. Pandemic-specific stressors related to economic hardship, social isolation, and health uncertainties can compound academic challenges associated with the transition to remote learning. Since transitioning to online learning, post-secondary students have reported increases in depressive and anxious symptoms (Fruehwirth et al., 2021) and lower psychological well-being (Dodd et al., 2021).

Emerging research has focused on academic challenges experienced by undergraduate students during COVID-19 pandemic. In a study of 114 undergraduate students, Giusti et al. (2021), found that experiences of challenges were mixed with 55% reported cognitive challenges related to concentration and learning abilities in attending online lessons, while 25% reported better concentration and learning abilities. Similarly, 60% of the students reported the lack of “face-to-face” contact with teachers as the main negative aspect, with 40% reporting difficulty interacting with teachers during the online lessons on the platform. Changes in study context and habits during the shift to online distance education increased the likelihood of poor perceived academic performance by almost four times. However, when attention and memory impairment, COVID anxiety and depression, and satisfaction with distance education were added to the model, changes in the study context and habits was no longer significant. Instead, reporting impairments in attention and concentration during distance education increased the likelihood of perceived poor academic performance by more than 8 times, and high COVID anxiety increased the perception of poor academic performance more than three times. Overall, satisfaction with distance education seemed to serve as a protective factor against perceptions of poor academic performance. Findings from this study were based on a small cross-sectional online convenience sample of university students at one university in Italy.

Negative associations between online learning and academic engagement during the pandemic have been reported in a sample of Canadian undergraduate students. Daniels et al. (2021) found online learning during the pandemic was associated with lower achievement goals, school engagement, and perceptions of success. While, diminished school engagement can indirectly impact academic performance, further research is needed to contribute to a more nuanced explanation of the underlying mechanisms that impact student success to further support students faced with the challenges of learning fully online during the pandemic.

Theoretically, challenges create opportunities for students to exercise self-regulatory control by deploying and adapting strategies and practices for the academic challenges they face. In other words, SRL practices provide mechanisms for productively responding to academic challenges and potentially buffering the effect of increased COVID-related stress that occurred after the shift to fully online undergraduate instruction. Further research is needed to understand (a) the kinds of challenges impacting student success and perceptions of success during the pandemic, (b) the impact of pandemic related distress on academic performance, and (c) the role of SRL practices in mitigating the impact of those challenges on academic performance. In this study, we focus specifically on academic challenges that impede students’ metacognitive and motivational abilities.

### Impact of Self-Regulated Learning Instruction/Intervention for Academic Success

Providing instruction about SRL through SRL interventions and courses has been found to improve academic performance, strategy engagement, and motivation (Jansen et al., 2019; Theobald, 2021), even when instructional prompts are provided without direct strategy instruction (Bannert and Reimann, 2012). Meta-analysis results indicate the effect of SRL interventions on academic achievement is partially mediated by SRL practices (Jansen et al., 2019). SRL instruction has been found to influence metacognitive and resource management strategy use, with smaller effects on cognitive strategy use (Theobald, 2021). Evidence suggests SRL interventions improve goal setting and planning strategies before learning as well as self-monitoring during studying, therefore Theobald (2021) recommended that future SRL training and research emphasize the role of metacognitive processes in improving strategy deployment and influences on academic achievement. In addition, Theobald’s (2021) meta-analysis revealed limitations in both the range and scope of self-report measured used to examine SRL practices.

Despite evidence that SRL strategy use is associated with higher academic achievement in online learning courses (Broadbent and Poon, 2015), research has rarely examined the influence of semester long SRL instruction/intervention on online academic achievement. Specifically, can online SRL training/instruction mitigate some of the impacts of pandemic-related distress on academic challenges and outcomes experienced by undergraduate students?

There is little question that the global pandemic dramatically changed the learning modes and experiences of undergraduate students. A rapid and unexpected shift to online learning can potentially introduce mounting new challenges and stressors for learners. Research is needed to understand the impact of pandemic-related stressors and academic challenges on academic performance. Furthermore, research is needed to examine the role of SRL intervention and practices in ameliorating the impact of these challenges on academic performance.

### Purpose and Hypotheses

The purpose of this study was to examine the role of SRL practices and SRL intervention in mitigating the impact of COVID-related psychological distress on academic success during fully online pandemic teaching. First, we tested our hypothesis that SRL practices would moderate the impact of COVID-related psychological distress and academic challenges (metacognitive and motivational) on academic performance for undergraduate students learning fully online during the pandemic (*N* = 496). Second, we tested our hypothesis that SRL training would moderate the impact of COVID distress on academic performance for first year undergraduate students. Finally, we examined differences in academic challenges and SRL practices reported by first year undergraduate students who did or did not receive online SRL intervention throughout their first semester of university to better understand some of the specific ways study practices and challenges may be experienced by students who receive SRL training (*n* = 157, SRL training group = 71; without training group = 86).

## Materials and Methods

### Sample

Participants (*N* = 463) were enrolled in a Western Canadian university and recruited from: (a) an elective learning-to-learn course in educational psychology designed to teach and promote strategic SRL processes and strategies (*n* = 82), and (b) a psychology research participation pool open to students from all faculties who enrolled in at least one undergraduate psychology course (*n* = 381). Most students (45%) were registered with the Faculty of Social Sciences with most (41%) majoring in psychology. Introduction to Psychology (PSYC 100A) is a required or recommended course across several faculties and programs at this university. Students enrolled in the elective course completed questionnaires as part of course activity requirements. Students from the SONA psychology research participation pool received one course credit that could be allocated to any of their psychology courses for participating in the research. All participants gave consent to participate in research and received debriefing about their SRL practices and challenges. All students attended university fully online for that academic year. Prior to the pandemic, online learning was not a typical mode of instruction at this university. While students may have had experience with one distance course during high school, few students had pre-pandemic experience with fully online instruction across all courses.

### Self-Regulated Learning Instruction

Self-regulated learning instruction was provided in a credit-bearing first year undergraduate educational psychology course (*ED-D101: Learning strategies for University Success)* introducing the science behind learning and motivation from an SRL perspective. The course consisted of a series of eleven online learning modules delivered over one academic semester (13 weeks).

Content for each weekly learning module was delivered asynchronously online through D2L Brightspace, 2020^1^. Students started by reading an interactive chapter that included text, video, images, and examples. Every topic was introduced with a link to the process of self-regulating learning to manage day-to-day academic challenges. Weekly topics included: (1) Introduction to online learning, (2) Introduction to SRL and academic success, (3) Understanding academic tasks and expectations, (4) Setting goals and monitoring learning during study sessions, (5) Information processing and active cognitive processes (e.g., activating prior knowledge, generative processing, etc.), (6) Understanding and regulating motivation, (7) Regulating time and procrastination, (8) Reading, notetaking, and learning in reading the social sciences, (9) Reading, notetaking and learning computational and STEM related concepts and procedures, (10) Regulating emotions, mental health, and well-being, (11) Active rehearsal and exam preparation.

The asynchronous modules were coupled with weekly synchronous online small-group lab sessions delivered through *Zoom* (Zoom Video Communications Inc, 2016) and coupled with *Microsoft-365 Teams* channels for communicating and sharing resources and examples. Each online lab engaged 20 students and a teaching assistant in guided activities and discussions designed to build on the content module by applying strategies to students undergraduate courses and respective disciplinary areas.

Students also accessed an online Strategy Library stocked with over 100 strategies organized by factors contributing to student success (Hadwin, 2020). Each strategy included a description, introduction to the science about why it works, instructions about how to use the strategy and examples of that strategy in use. The strategy library was created and deployed through D2L Brightspace for all students at the University.

A key component of this course was developing metacognitive awareness of studying approaches, strategies, and outcomes. Students completed a weekly study diary activity (McCardle and Hadwin, 2015) requiring them to (a) plan a 1-to-2-hour study session for one core academic course by setting a specific learning goal, (b) completing that study session, and (c) reflecting on the outcomes, experiences and challenges encountered during that study session. This weekly activity intentionally engaged students in cycles of SRL with the goal of helping them leverage past experiences to optimize subsequent study sessions.

### Procedure

Surveys were administered in week 11 of a 13-week academic semester in the Fall of 2020 during the COVID-19 pandemic. Immediately after completing the questionnaire, students were provided with individualized profile reports and provided with instruction about how to use those reports to self-diagnose strengths and weaknesses in studying and identify strategies for improving SRL based on their individualized data. Students were provided links to specific evidence-based study strategies based on those individualized reports.

### Measures

***The self-regulated learning profile and self-diagnostic instrument*** (SRL-PSD; Hadwin et al., 2021) examines self-reported SRL practices and SRL challenges (see Appendix 1 for items in the scales). The scale was developed to incorporate practices associated with forethought (task understanding, goal setting, and planning), performance, and reflection during studying. Importantly, the scale includes practices associated with metacognitive adaptation during studying that are often under-examined in SRL research.

***The SRL- Practices Scale*** (SRL-P; Hadwin et al., 2021) measures students’ perceptions about their engagement in practices that foster SRL. The SRL-P comprises 31 items yielding 8 subscales related to (1) task understanding (e.g., “*Asked myself if I know what is important to learn*”), (2) goal management (e.g., “*Set goals for my work*”), (3) motivation: task value (e.g., “*Reflected on why this work is important*”), (4) motivation: appraisal (e.g., “*Assessed if think I can do it*”), (5) time management (e.g., “*Created a timeline or schedule*), (6) metacognitive monitoring (e.g., “*Asked myself if I am understanding the material*”), (7) metacognitive adaptation (e.g., “*Modified my plans for the task*”), and (8) social engagement (e.g., “*Got to know people in the class*”). Participants were required to rate their level of agreement for each statement in a five-point Likert scale ranging from (1) Strongly disagree to (5) Strongly agree.

***Self-regulated learning challenges scale*** (SRL-C; Hadwin et al., 2021) is comprised of 43 items (5 subscales) assessing the degree to which students encountered a range of challenges in their studying. Responses were reported on a 5-point Likert scale from (1) *Strongly disagree* to (5) *Strongly agree*. Higher scores indicate a student is struggling to manage aspects of studying theoretically and empirically associated with student success and performance. The *Motivation challenge* subscale was comprised of six-items related to motivational beliefs, interest, and persistence. The *Metacognitive challenge* subscale was comprised of 10-items related to task understanding, goal setting, planning, and monitoring. The *Cognitive challenge* subscale was comprised of eight-items related to attending, encoding, and remembering. The *Behavioral challenge* subscale was comprised of eight-items related to managing time, tasks, and procrastination. The *Socio-emotional challenge* subscale was comprised of 11-items related to emotional, social, and relational aspects of academic success.

***Academic Performance*** was measured using two distinct but correlated variables: self-reported *perceptions of academic performance* where students were asked to report on what overall GPA (average grade) they expected to get this year and objective measures of academic performance obtained through institutional reports (i.e., GPA on a 9-point scale).

***COVID-19 Distress*** was adapted from the COVID Stress Scales (Taylor et al., 2020) which comprised of 23 items related to (1) danger and contamination fears (e.g., I am worried about catching the virus from handling money or using a debit machine), (2) fears about economic consequences (e.g., “I am worried my financial situation will be affected by COVID-19”), (3) compulsive checking and reassurance seeking (e.g., “I sought reassurance from friends and family about COVID-19”), and (4) traumatic stress symptoms related to the pandemic (e.g., “Disturbing mental images about the virus popped into my head against my will”). In addition, two items related to feelings of guilt and shame about the pandemic (“I feel guilty for not doing more to prevent COVID-19”; “I feel ashamed of my emotional reactions to COVID-19”) were also added from the COVID-19 Peritraumatic Distress Index (CPDI; Qiu et al., 2020). The items were rated on a 5-point scale ranging from 0 (not at all) to 4 (extremely) and were summed to create a composite for COVID-19 rumination.

### Data Analytic Strategy

**FIGURE 1:**
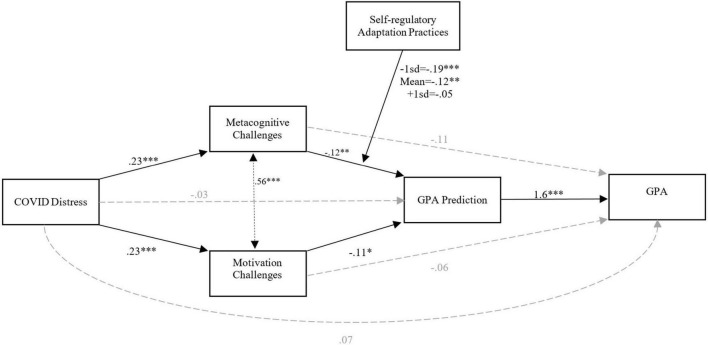
Structural relationship between COVID-D (predictor), metacognitive and motivation challenges (parallel mediators), GPA-P (serial mediator), adaptation practices (moderator), and GPA (outcome): a conditional process path analysis (*N* = 463).

Conditional process analysis was used to test the hypotheses (Hayes, 2015, 2017) using Process syntax version 3.5.3. As shown in Figure 1, COVID-D was regressed on the GPA (the outcome variable) with two parallel mediators (metacognitive and motivation challenges) and one serial mediator (perceptions of GPA; GPA-P). Adaptation practices was added to the model to test if the magnitude of COVID-D’s indirect effect on GPA-P through metacognitive challenges depends on variation in adaptation practices scores. It was hypothesized that experiencing higher COVID-D would increase students’ metacognitive and motivational challenges. These challenges, in turn, would have an inverse effect on students’ perception of academic performance which in turn diminishes their actual academic performance. This model is regarded as an inconsistent mediation model (MacKinnon et al., 2007) because it includes mediated effects with different signs.

## Results

### The Role of Self-Regulated Learning Practices in Moderating the Impact of COVID Distress on GPA

Descriptive statistics and intercorrelations for the main study variables for the entire sample are provided in Tables 1, 2. As shown in Table 2, academic challenges negatively associated with GPA prediction and GPA. While COVID distress was not associated with GPA, it was found to be negatively related to GPA predictions (GPA-P). Results from the model represented in Figure 1 indicate COVID distress positively predicts metacognitive challenges [*B* = 0.23, *t*(461) = 4.96, *p* < 0.001] as well as motivation challenges [*B* = 0.23, *t(*461) = 5, *p* < 0.001]. These challenges both have inverse effects on GPA prediction. The inverse effect indicates higher levels of challenge were associated with lower GPA prediction [*B_Metacognition_* = –0.12, *t*(457) = –2.61, *p* = 0.009 | *B_Motivation_* = –0.11, *t*(457) = –2.47, *p* = 0.014]. Adaptation was added as a moderator to test if the indirect effect of COVID-D on GPA prediction (GPA-P) through metacognitive challenges is contingent on adaptation practices. The interaction of metacognitive challenges and adaptation practices was a significant predictor of students’ GPA prediction [*B* = 0.72, *t*(457) = 2.3, *p* = 0.020]. Further analysis of conditional effects of the metacognitive challenges at values of the adaptation practices showed that the inverse indirect impact of COVID-D on GPA-P is stronger for students who practiced less adaptation. To elaborate, while this effect is significant at 1 standard deviation below adaptation average [*B* = –0.19, *t*(457) = –3.53, *p* = 0.0005], it is less strong at the adaption average value [*B* = –0.12, *t*(457) = –2.6, *p* = 0.009], and less strong and non-significant at 1 standard deviation above the adaptation average [*B* = –0.05, *t*(457) = –0.89, *p* = 0.37]. The index of moderated mediation (difference between conditional indirect effects, see Hayes, 2015) was also significant (Index = 0.026, Boot*SE* = 012, 95% Boot*CI* [0.003, 0.52]) indicating higher versus lower levels of adaptation practices produce a differential effect on the indirect effect of COVID-D on GPA-P through metacognitive challenges. Follow up Johnson–Neyman significance region(s) analysis showed that the effect is significant for adaptation values falling above 61 percentiles. Finally, GPA-P was shown to have a strong direct influence on GPA [*B* = 1.6, *t(*458) = 19.23, *p* < 0.001].

**TABLE 1:** Descriptive statistics for the whole sample.

	Scale variable (sub-scale)	Mean	*SD*
–	High School GPA	87.74	6.05
–	Term GPA	6.68	1.82
–	GPA-P	3.45	0.78
–	COVID-D	–0.04	0.99
SRL-P	Goal management	0.09	1.00
SRL-P	Task understanding	0.03	0.99
SRL-P	Task value	–0.05	1.00
SRL-P	Motivation appraisal	0.04	0.98
SRL-P	Adaptation	0.00	1.01
SRL-P	Monitoring	0.07	0.94
SRL-P	Social engagement	0.00	1.01
SRL-P	Time management	0.08	1.00
SRL-C	Metacognition challenges	–0.09	0.98
SRL-C	Socio-emotional challenges	–0.01	0.98
SRL-C	Cognitive challenges	–0.08	1.01
SRL-C	Initiating and sustaining engagement challenges	–0.01	0.98
SRL-C	Goal and time management challenges	–0.10	1.00
SRL-C	Motivation challenges	–0.05	1.00

*SRL, self-regulated learning; P, SRL practices; C, SRL challenges.*

**TABLE 2:** Intercorrelations for study variables (*N* = 463).

Sub-scales	GPA TERM	GPA-P	COVID-D	Adaptation (SRL-P)	Metacognition (SRL-C)	Social and emotional (SRL-P)	Cognitive (SRL-C)	Initiating and Sustaining engagement (SRL-C)	Goal and time management (SRL-C)	Motivation (SRL-C)
Term GPA	1									
GPA-P	0.691**	1								
COVID-D	–0.054	−0.104*	1							
Adaptation (SRL-P)	–0.090	–0.029	0.048	1						
Metacognition challenges	−0.226**	−0.248**	0.225**	0.020	1					
Social and emotional challenges	0.046	–0.033	0.299**	0.003	0.514**	1				
Cognitive challenges	−0.255**	−0.236**	0.148**	0.031	0.639**	0.354**	1			
Initiating and sustaining engagement	–0.073	−0.121**	0.116*	–0.040	0.429**	0.353**	0.340**	1		
Goal and time management	−0.148**	−0.105*	0.058	0.064	0.571**	0.344**	0.464**	0.546**	1	
Motivation challenges	−0.235**	−0.245**	0.226**	0.033	0.613**	0.489**	0.594**	0.472**	0.536**	1

*SRL-P, SRL Practices; SRL-C, SRL Challenges.*

*p ≤ 0.05, **p ≤ 0.01.

COVID-D was found to have no significant direct effect (*c’*) on GPA [*B* = 0.07, *t*(458) = 0.104, *p* = 0.30]. Analysis of indirect effects showed that motivation and metacognitive challenges fully mediated the effect of COVID-D on GPA-P, making the direct link between COVID-D and GPA-P insignificant [*B* = –0.03, *t(*457) = –0.75, *p* = 0.45]. Also, COVID-D was found to have no significant indirect effect on GPA through motivation challenges (*B* = –0.026, Boot*SE* = 0.019, 95% BootCI [–0.065,0.01]), metacognition challenges (*B* = –0.01, *BootSE* = 0.02, 95% Boot*CI* [–0.05,0.02]), and GPA-P (*B* = –0.045, Boot*SE* = 0.068, 95% Boot*CI* [–0.17,0.09]).

Table 3 contains the indirect effects tested in model 1. Rows 4 and 5 in the table represent two statistically significant processes by which COVID distress indirectly influences academic performance, i.e., through enhancing academic challenges, which in turn decreases GPA prediction and GPA itself [*F(*4,458) = 107.14, *p* < 001, *R^2^* = 0.48]. The indirect effect through metacognitive challenges (Table 3, row 5) is presented at three levels of adaptation practices to reflect its moderating role in the model.

**TABLE 3:** Unconditional and conditional indirect effects of COVID-D on GPA.

										Bootstrap		
									Effect	*SE*	LLCI	ULCI
1	COVID-D	→	MOT CH	→	GPA				–0.026	0.019	–0.065	0.01
2	COVID-D	→	META CH	→	GPA				–0.014	0.02	–0.055	0.024
3	COVID-D	→	GPAP	→	GPA				–0.045	0.068	–0.17	0.09
4	COVID-D	→	MOT CH	→	GPAP	→	GPA		–0.04	0.02	–0.086	–0.007
5								ADAPT SD-1	–0.07	0.024	–0.12	–0.026
	COVID-D	→	META CH	→ **→** ADAPT	GPAP	→	GPA	ADAPT Average	–0.043	0.019	–0.085	–0.009
								ADAPT SD+1	–0.018	0.022	–0.064	0.023

*COVID-D, COVID distress; MOT CH, motivational challenges; META CH, metacognitive challenges; GPAP, GPA prediction; ADAPT, adaptation practices.*

### Self-Regulated Learning Training as a Moderator of the Impact of COVID Distress on GPA for 1st Year Students

**FIGURE 2:**
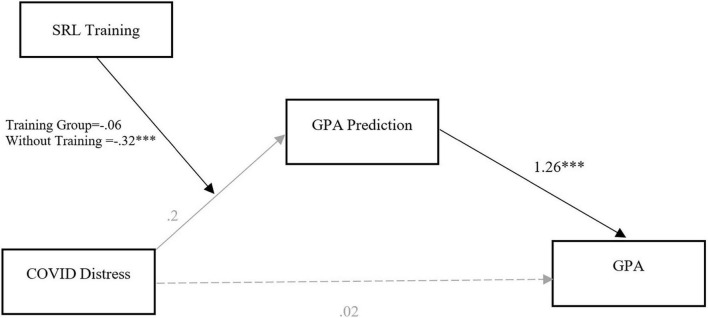
SRL training as a moderator of the impact of COVID distress on GPA for 1st year students: a moderated mediation path analysis (*N* = 169).

A mediated moderation model was used to test if SRL training moderates the impact of COVID distress on academic performance. Analysis was conducted on a subset of the sample comprised of 1st year students as only first-year students participated in the SRL intervention (*n* = 157, SRL training group = 71; without training group = 86; means and standard deviations are provided in Table 4). Parameters were estimated with a robust standard error (HC3 method; Davidson and MacKinnon, 1993) to obtain unbiased standard errors under heteroscedasticity. As depicted in Figure 2, results show that COVID-D had no significant influence on GPA-P [*B* = 0.20, *t*(152) = 1.08, *p* = 0.28] but the interaction of training (0, 1) and COVID-D negatively impacted GPA-P [*B* = 0.26, *t*(152) = –2.15, *p* = 0.03]. Conditional effects of the COVID-D across two groups indicate that COVID-D negatively affected students’ GPA predictions in the group with no SRL training [*B* = –0.32, *t*(152) = –3.6, *p* < 0.001] but such an effect was not observed in the group that had received SRL training [*B* = –0.06, *t*(52) = –0.69, *p* = 0.5]. Consistent with the results of the previous model, GPA-P had a strong positive influence on GPA [*B* = 1.26, *t*(153) = 7.6, *p* < 0.001]. The direct path in the model (*C’*) is not significant [*B* = 0.05, *t*(153) = 0.33, *p* = 0.74] meaning that COVID-D impacts GPA only indirectly through mediation of GPA-P in the model [*F*(2,153) = 37.13, *p* < 0.001, *R*^2^ = 0.27].

**TABLE 4:** Descriptive statistics for the “first-year students” sub-sample (Model 2).

Scale Variable (Sub-scale)			Mean	*SD*
–	High School GPA	G1	88.05	5.28
		G2	90.88	4.97
–	Term GPA	G1	5.92	1.63
		G2	6.69	1.85
–	GPA-P	G1	3.10	0.62
		G2	3.51	0.81
–	COVID-D	G1	–0.19	0.98
		G2	–0.11	0.95
SRL-P	Goal management	G1	0.18	0.76
		G2	0.15	1.06
SRL-P	Task understanding	G1	0.00	0.85
		G2	0.21	0.95
SRL-P	Task value	G1	0.09	0.93
		G2	–0.04	1.05
SRL-P	Motivation Appraisal	G1	–0.14	0.99
		G2	0.19	0.96
SRL-P	Adaptation	G1	0.27	0.92
		G2	0.07	0.95
SRL-P	Monitoring	G1	0.20	0.83
		G2	0.19	0.86
SRL-P	Social engagement	G1	0.15	0.98
		G2	–0.05	1.06
SRL-P	Time management	G1	0.09	1.00
		G2	0.15	1.02
SRL-C	Metacognition Challenges	G1	–0.39	0.75
		G2	–0.02	1.04
SRL-C	Socio-emotional Challenges	G1	–0.44	0.90
		G2	0.02	1.02
SRL-C	Cognitive Challenges	G1	–0.33	0.83
		G2	–0.03	1.06
SRL-C	Initiating and Sustaining engagement Challenges	G1	–0.15	0.87
		G2	–0.15	1.13
SRL-C	Goal and Time Management Challenges	G1	–0.20	0.83
		G2	–0.24	1.11
SRL-C	Motivation Challenges	G1	–0.27	0.85
		G2	0.01	1.09

*Training Group = (G1, n = 71); Without Training = (G2, n = 86); COVID- Distress, and all SRL-P and SRL-C dimensions are Croon’s Bias-Corrected Bartlett factor scores. GPA-P, GPA perception; COVID-D, COVID distress; SRL-P, SRL practices; SRL-C, SRL challenges.*

The index of moderated mediation was found to be significant (Index = –0.33, Boot*SE* = 0.16, 95% Boot*CI* [–0.65, –0.006]) showing that the full mediated effect between COVID-D and GPA through GPA-P significantly differs across the two groups. Investigating the indirect effects in the model shows that among those who did not receive SRL training, COVID-D had a significant negative indirect effect on GPA through GPA-P (*B* = –0.4, Boot*SE* = 0.13, 95% Boot*CI* [–0.64, –0.13]). In contrast, this indirect effect was weaker and non-significant in the SRL training group (*B* = –0.07, Boot*SE* = 0.11, 95% Boot*CI* [–0.29,0.14]). In sum, findings support that SRL training moderated the impact of COVID distress on academic performance such that the effect of COVID distress on academic performance through GPA prediction was mollified for students who were enrolled in ED-D101 compared to students who were not enrolled in ED-D101.

### Mean Differences in Self-Reported Academic Challenges and Self-Regulated Learning Practices Between 1st Year Students With and Without Self-Regulated Learning Training

Two multivariate analyses of covariance (MANCOVA) were conducted to better understand how first-year students with and without SRL training differed in terms of academic challenges and SRL practices after controlling for their incoming GPA. Box’s test of equality of covariance matrices was insignificant for both academic challenges and SRL practices [for Academic Challenges: *M* = 32.7, *F* = (21,81859) = 1.5, *p* = 0.07; for SRL practices: *M* = 28.9, *F*(36,75006) = 0.76, *p* = 0.85] indicating that the observed covariance matrices of the dependent variables are equal across groups. Homogeneity of regression slopes was tested by adding interaction terms between the covariate and independent variable into analyses and making sure the term is insignificant.

Results summarized in Table 5 showed that while there was a statistically significant difference between the groups with and without SRL training on the combined academic challenges after controlling for incoming GPA [*F*(6,148) = 3.2, *p* = 0.005, Wilks’ Λ = 0.885,$\eta_{\text{p}}^{2}$ = 0.115], there was no statistically significant difference between the groups on the combined SRL practices scales after controlling for incoming GPA [*F*(8,147) = 1.55, *p* = 0.144, Wilks’ Λ = 0.92, $\eta_{\text{p}}^{2}$ = 0.078].

**TABLE 5:** Pairwise comparisons of SRL-P and SRL-C based on estimated marginal means (SRL Training = I, Without Training = J).

	Sub-scale	Mean difference (I-J)	Robust standard error (HC3)	*t*	Sig.	95% CI for difference		$\eta_{\text{p}}^{2}$	Levene’s test *F*(1,155)
Scale						LL	UL		
SRL-C	Metacognition challenges	–0.42	0.149	–2.86	0.005	–0.72	–0.13	0.050	12.66***
SRL-C	Cognitive challenges	–0.47	0.158	–2.96	0.004	–0.72	–0.16	0.054	3.93*
SRL-C	Motivation challenges	–0.37	0.167	–2.23	0.027	–0.70	–0.04	0.031	5.64*
SRL-C	Social and emotional challenges	–0.43	0.165	–2.6	0.010	–0.76	–0.10	0.042	0.25
SRL-C	Initiating and sustaining engagement challenges	–0.05	0.174	–0.27	0.789	–0.39	0.3	0.000	6.3*
SRL-C	Goal and time management challenges	0.00	0.166	0.02	0.983	–0.32	0.33	0.000	7.16**
SRL-P	Goal management	–0.002	0.146	–0.014	0.989	–0.291	0.287	0.000	9.4**
SRL-P	Task understanding	–0.121	0.148	–0.822	0.412	–0.413	0.170	0.004	0.16
SRL-P	Task value	0.181	0.165	1.101	0.273	–0.144	0.506	0.008	2.26
SRL-P	Motivation appraisal	–0.333	0.167	–1.987	0.049	–0.663	–0.002	0.025	0.09
SRL-P	Adaptation	0.160	0.159	1.009	0.315	–0.154	0.474	0.007	0.14
SRL-P	Monitoring	0.047	0.148	0.317	0.751	–0.245	0.339	0.001	0.71
SRL-P	Social engagement	0.194	0.163	1.186	0.237	–0.129	0.516	0.009	0.37
SRL-P	Time management	–0.053	0.174	–0.306	0.760	–0.397	0.291	0.001	0.03

*SRL-P, SRL practices; SRL-C, SRL challenges.*

*p ≤ 0.05, **p ≤ 0.01, ***p ≤ 0.001.

Follow-up univariate analyses were carried out to elaborate more on adjusted mean differences between the groups in terms of each dependent variable after controlling for the effect of incoming GPA. As we had unequal variances particularly for SRL practices scales, we used HC3 robust standard error estimator to handle the heteroscedasticity problem that might have caused by heterogeneity of error variances across the two groups. However, research indicates that univariate group analyses are robust to moderate violations of homogeneity of variances if group sample sizes are approximately equal (Nimon, 2012). This analyses revealed that after controlling for the effect of incoming GPA, students without SRL training had a significantly higher average adjusted mean for the SRL practice – motivation appraisal scale [Mean Difference = 0.33, *t*(154) = –1.99, *p* = 0.049, $\eta_{\text{p}}^{2}$ = 0.025], metacognitive challenges [Mean Difference = 0.42, *t*(154) = –2.86, *p* = 0.005, $\eta_{\text{p}}^{2}$ = 0.05], cognitive challenges [Mean Difference = 0.47, *t*(154) = –2.96, *p* = 0.004, $\eta_{\text{p}}^{2}$ = 0.054], motivation challenges [Mean Difference = 0.37, *t*(154) = –2.23, *p* = 0.027, $\eta_{\text{p}}^{2}$ = 0.031], and socio-emotional challenges [Mean Difference = 0.43, *t*(154) = –2.6, *p* = 0.01, $\eta_{\text{p}}^{2}$ = 0.042].

To summarize, students without SRL training reported higher levels of social-emotional, cognitive, metacognitive, and motivation challenges than students with SRL training. Furthermore, students with SRL training reported fewer motivation appraisal practices such as thinking about why they are being asked to know a concept, reflecting on why it is important, and making judgments about the usefulness or value of the content.

## Discussion

The current study examined how pandemic-related stressors affect students’ academic performance and the role of SRL practices in ameliorating the impact of academic challenges on performance. Findings show that COVID stressors impaired students’ academic performance through introducing more metacognitive and motivational academic challenges. However, for individuals who are highly skilled in SRL practices, specifically around adaptation (i.e., scoring in the top third of the sample on the SRL adaptation measure), academic challenges did not mediate the relationship between COVID stressors and academic performance. This finding is consistent with SRL theory which posits that self-regulated learners who are more adaptive fare better in the face of ongoing academic adversities because they can recognize that the acquisition of learning skills requires systematic variations in approaches that will help them overcome learning difficulties (Winne and Hadwin, 2008; Zimmerman, 2008). Moreover, students with a more adaptive profile of SRL strategy usage tend to report higher academic achievement (Liu et al., 2014).

While academic challenges are normative for students, the abrupt shift to remote learning coupled with impeding psychological distress about the pandemic can further tax already compromised SRL abilities. Pandemic-related stressors can exacerbate existing academic challenges particularly among those who struggle to monitor and adapt their learning strategies effectively to changing demands. The enhanced process of self-reflection and adaptive decision-making among self-regulated learners requires a greater understanding and awareness of one’s own learning processes, or meta-cognitive ability. While few studies have directly examined SRL practices specifically in the context of adaption, meta-cognitive awareness has been linked to academic performance (e.g., Ward and Butler, 2019). Consistent with the SRL process model (Zimmerman, 2000), regular use of forethought (e.g., strategic planning, goal setting) and self-reflection seems to distinguish high achieving from poor achieving students, enabling them to adapt more effectively to shifting contextual changes. Indeed, high-achieving students tend to use higher-quality learning strategies related to forethought and self-reflection (Colthorpe et al., 2019).

Our findings also show that students’ SRL practices can be supported through a course-based intervention that help students navigate academic challenges associated with online learning. Specifically, first year students who received SRL training in the course *ED-D101: Learning Strategies for University Success* reported fewer metacognitive, cognitive, motivational, and socio-emotional challenges compared to their counterparts who were not enrolled in the SRL course. Moreover, the intervention moderated the impact of pandemic-related stressors on academic performance since the negative impact of COVID stressors on academic performance was stronger among students who were not enrolled in the SRL course. This finding suggests that students who are taught strategic ways to enhance their SRL through understanding tasks, setting goals, monitoring their studying approaches, and managing their time, and mental-health and well-being are better able to overcome academic challenges compared to students without this SRL-focused training.

Leveraging SRL instructional tools and resources can promote metacognitive monitoring processes. SRL interventions have shown efficacy in improving academic performance, in part, through enhancing their knowledge of SRL and engagement in SRL activities (de Bruijn-Smolders et al., 2016; Jansen et al., 2019). Guiding students to better assess their learning gains, attributing those gains to a specific effective strategy, and systemically applying that strategy to appropriate tasks, optimizes their learning. Moreover, encouraging students to identify and modify strategies that are not working enhances their adaptive ability. The iterative cycle of monitoring tasks and recalibrating strategies is not intuitive for all students particularly under competing demands of academic and social post-secondary life. Compounding such demands with unique circumstances surrounding a global pandemic can further impede students’ ability to transfer their self-regulatory abilities across multiple contexts. For example, studying habits that were effective for an in-person learning may not be optimal in a online learning. SRL is contextual and dynamic, requiring learners to regulate their cognition, motivation and emotions during learning tasks that change across different situations (Winne and Hadwin, 2008; Zimmerman, 2008). Changing tasks and demands introduce new challenges overcomeing these challenges requires learners to successfully adapt and adjust previously learned strategies. During the pandemic, post-secondary students encountered new academic challenges necessitating SRL capacities (e.g., metacognitive awareness); students with fewer SRL skills are less equipped to handle pandemic-related stressors that diminish academic performance. However, our findings suggest that students provided with SRL training were able to moderate the impact of COVID distress on academic performance, mollifying its effect. Providing students with the opportunity to reassess learning approaches and identify specific challenge areas through SRL interventions enables them to strategically direct studying efforts. For example, learning task understanding skills for deciphering specific course content and assigned tasks more accurately is an important predictor of academic performance (Oshige et al., 2007; Hadwin et al., 2008). Promoting students’ awareness of their own learning and guiding them to use higher-quality strategies related to goal setting, strategic planning and self-evaluation can improve student learning outcomes (Colthorpe et al., 2019). However, it is worth noting that while the SRL intervention group reported fewer academic challenges compared to the non-intervention group, they did not differ significantly in their levels of SRL practices after controlling for incoming GPA (except for motivation appraisal). It may be that the SRL intervention operates through enhancing students’ pre-existing SRL skills by helping students apply learning strategies more efficiently and adaptively, thereby mitigating academic challenges.

### Limitations and Future Direction

While the current study offers a more nuanced understanding of how SRL processes mitigate academic challenges in the face of a contextual stressor, such effects are limited to the specific context of the 2020 COVID-19 pandemic. Nevertheless, the pandemic provides a unique examination of the unprecedented changes to students’ educational environments that are not typical of everyday academic challenges. Individual differences in the SRL skills students’ use to overcome these academic challenges can help educators support the transition from varying modes of instruction (e.g., online/blended/in-person settings). Another limitation of the study surrounds the use of self-reports of SRL practices which can be subjected to respondent and recall biases. Moreover, as SRL is a context-dependent process, SRL practices can change depending on that task and goal at hand which may not be accurately captured in traditional self-reported inventories. Nonetheless, self-report still provides valuable insights into learner’s motivation processes and perceptions of how they monitor, set goals, and adapt effectively particularly if measures are sensitive to time and task (McCardle and Hadwin, 2015). More research may benefit from using objective measures of SRL practices to discern the frequency and quality of these skills (e.g., Winne, 2010). Lastly, it is important to acknowledge limits to the design of the study. Specifically, participants were not randomly assigned to the intervention condition, which limits the comparability across groups. As group comparisons were only permitted among first year students in the analysis (i.e., to match the samples), such truncation significantly reduced the sample size. Without a true randomized control trial (RCT) design with pre-test and post-test differences in both groups, findings regarding the effectiveness of the intervention should be viewed with caution. RCTs of SRL interventions are needed to systematically evaluate its impact on student success.

## Conclusion

The prolonged COVID-19 pandemic continues to impact post-secondary institutions across the globe. Shifts to online instruction in the Fall of 2020 combined with the ongoing demand for instructional approaches that flexibly blend online options with on-campus delivery amplifies the need for students to develop and deploy SRL practices. Findings from this study demonstrate that psychological stressors related to the pandemic can impair students’ academic performance by introducing more metacognitive and motivational challenges during online learning. However, self-regulatory learning practices that promote adaption to new learning contexts, tasks and situations can help alleviate the impact of COVID stressors on academic performance. Moreover, students SRL skills can be bolstered by a 13-week on online academic course that explicitly supports students to diagnose and overcome a range of academic difficulties associated with online learning.

While the COVID-19 pandemic will end, it has illuminated the effect that additional stressors have on post-secondary students particularly when they are transitioning to first year studies and new modes of delivery such as online learning. Findings from this study point to the critical importance of equipping students with a toolbox of self-regulatory practices and strategies that help them to adaptively respond to new live and school stressors. Findings from this study indicate that learning to manage stressors and remediate academic challenges using self-regulatory strategies and practices is something that can (a) be taught over an academic semester, and (b) contribute to better academic outcomes such as GPA. Proactively investing in these types of courses may have potential to improve online learning outcomes and position institutions and students to adapt to future life stressors and global events more proactively.

## Data Availability Statement

The raw data supporting the conclusions of this article will be made available by the authors, without undue reservation.

## Ethics Statement

The studies involving human participants were reviewed and approved by University of Victoria Human Research Ethics Board. The patients/participants provided their written informed consent to participate in this study.

## Author Contributions

AH is the grant holder and contributed to the funding, theory, development and delivery of the SRL online intervention, formulation of study design and is the main writer of the submitted manuscript (i.e., the section “Introduction”). PS contributed to the evaluation funding, formulation of study design, theory, and manuscript preparations (e.g., writing of discussion). RR contributed to the study design, programming and coordination of the online evaluation, facilitated the SRL intervention, and conducted the statistical analyses. LB-O facilitated the SRL intervention, conducted the evaluation of the intervention, and made editorial contributions to the manuscript. All authors contributed to the article and approved the submitted version.

## Conflict of Interest

The authors declare that the research was conducted in the absence of any commercial or financial relationships that could be construed as a potential conflict of interest.

## Publisher’s Note

All claims expressed in this article are solely those of the authors and do not necessarily represent those of their affiliated organizations, or those of the publisher, the editors and the reviewers. Any product that may be evaluated in this article, or claim that may be made by its manufacturer, is not guaranteed or endorsed by the publisher.

## Footnotes

1
https://bright.uvic.ca/d2l/home/73882
