# Supplementary material for: Do Self-Regulated Learning Practices and Intervention Mitigate the Impact of Academic Challenges and COVID-19 Distress on Academic Performance During Online Learning?
Source: Front Psychol. 2022 Mar 16;13:813529. doi: 10.3389/fpsyg.2022.813529 (PMC8966875; doi:10.3389/fpsyg.2022.813529)
Supplement: Supplementary file 1 [file Table_1.DOCX]

**Appendix 1.**

**Table 1.**

*The Self-Regulated Learning Challenges Scale (SRL-C)*

| *SRP-C factors and Items* | |
| --- | --- |
| Metacognitive Challenges | **Met1**: Adjusting to new situations and contexts {*excluded}  **Met2**: Breaking tasks down  **Met3**: Interpreting tasks and expectations  **Met4**: Knowing how to tackle tasks  **Met5**: Knowing how or when to fix strategies/study skills  **Met6**: Knowing what questions to ask  **Met7**: Prioritizing or figuring out what is important  **Met8**: Monitoring my task or learning progress  **Met9**: Setting goals for studying  **Met10**: Applying the right strategies/techniques properly |
| Social and emotional challenges | **S-E1**: Feeling connected  **S-E2**:  feeling lonely  **S-E3**: Finding enjoyment at university  **S-E4**: Managing my emotions/feelings  **S-E5**: Managing relationships  **S-E6**: Taking care of my physical health [*Excluded]  **S-E7**: Taking care of my mental health and wellbeing |
| Cognitive Challenges | **Cog1**: Applying my learning  **Cog2**: Elaborating and connecting what I learned  **Cog3**: Grappling with language and communication  **Cog4**: Identifying important ideas  **Cog5**: Remembering things I learned  **Cog6**: Understanding learning materials and concepts |
| Initiating-sustaining engagement | **I-S1**: Wanting to do my schoolwork  **I-S2**:  putting things off (procrastinating)  **I-S3**: Focusing or concentrating  **I-S4**: Managing distractions |
| Behavioural Challenges  (Goal and time Management) | **Beh1**: Keeping my commitments or goals  **Beh2**: Organizing time and tasks  **Beh3**: Managing my time, tasks, or goals  **Beh4**: Meeting deadlines |
| Motivation Challenges | **Mot1**: Believing I can do my work  **Mot2**: Feeling like my work was worth doing  **Mot3**: Persisting when things got tough  **Mot4:** Being discouraged by setbacks [*added] |

| **Table 2**  *The Self-regulated Learning Practices Scale (SRL-P)* | |
| --- | --- |
| **Factors** | **Items** |
| Goal Management | **GM1**: Set goals for my work  **GM2**: Made goals to learn, understand, or remember  **GM3**: Set goals that will be useful for checking my progress  **GM4**: Identified specific content, ideas, or terms in my goals  **GM5**: Assessed my goal attainment |
| Task Understanding | **TU1**: Asked myself if I know what is important to learn  **TU2**: Made sure I understand terminology used in instructions  **TU3**: Thought about the professor's expectations and standards  **TU4**: Considered what knowledge or big ideas I should learn or demonstrate  **TU5**: Thought about relevant information and resources |
| Task Value | **TV1**: Thought about why we are being asked to know this stuff  **TV2**: Reflected on why this work is important  **TV3**: Made judgments about the usefulness or value of the content |
| Motivation Appraisal | **MA1**: Assessed if think I can do it  **MA2**: Assessed my feelings for the task  **MA3**: Evaluated the effort I was putting in |
| Monitoring | **Mo1**: Asked myself if I understand what I am supposed to be doing  **Mo2**: Asked myself if I was remembering  **Mo3**: Asked myself if I am understanding the material |
| Adaptation | **Ad1**: Changed my understanding of the task at hand  **Ad2**: Modified my plans for the task  **Ad3**: Switched to a different strategy or approach  **Ad4**: Changed my feelings about the task  **Ad5**: Altered the level of effort I put in  **Ad6**: Modified my beliefs about how well I would do my tasks |
| Time Management | **TM1**: Checked to see if I am staying on time  **TM2**: Chose goals that can be completed in one reasonable work session  **TM3**: Created a timeline or schedule |
| Social Engagement | **SE1**: Got to know people in the class  **SE2**: Had fun in online chats, discussions or via email with the instructor or other students  **SE3**: Helped classmates |
